# Growth Differentiation Factor-15 Is a Predictor of Mortality in Critically Ill Patients with Sepsis

**DOI:** 10.1155/2017/5271203

**Published:** 2017-10-18

**Authors:** Lukas Buendgens, Eray Yagmur, Jan Bruensing, Ulf Herbers, Christer Baeck, Christian Trautwein, Alexander Koch, Frank Tacke

**Affiliations:** ^1^Department of Medicine III, RWTH-University Hospital Aachen, Pauwelsstrasse 30, 52074 Aachen, Germany; ^2^Medical Care Center, Dr. Stein and Colleagues, 41061 Mönchengladbach, Germany

## Abstract

Growth differentiation factor-15 (GDF-15) is a member of the transforming growth factor-*β* superfamily related to inflammation and macrophage activation. Serum concentrations of GDF-15 can predict poor survival in chronic diseases, but its role in sepsis is obscure. Therefore, we investigated GDF-15 as a prognostic biomarker in critically ill patients. We measured GDF-15 levels in 219 critically ill patients (146 with sepsis, 73 without sepsis) upon admission to the intensive care unit (ICU), in comparison to 66 healthy controls. GDF-15 levels were significantly increased in ICU patients compared to controls. GDF-15 was further increased in sepsis and showed a strong association with organ dysfunction (kidney, liver and lactate) and disease severity (APACHE II and SOFA score). High GDF-15 concentrations at admission independently predicted ICU (HR 3.42; 95% CI 1.33–8.78) and overall mortality (HR 2.02, 95% CI 1.02–3.88) in all ICU critically ill patients as well as in a large subgroup of sepsis patients (ICU mortality: HR 3.16; 95% CI 1.10–9.07; overall mortality: HR 2.62; 95% CI 1.14–6.02). Collectively, serum GDF-15 levels are significantly increased in critically ill patients, associated with sepsis, organ failure, and disease severity. High GDF-15 levels at ICU admission predict short- and long-term mortality risk.

## 1. Introduction

Sepsis, severe sepsis, and septic shock are characterized by an organ dysfunction of varying degrees caused by a dysregulated immune response to an infection [1]. Sepsis poses—despite advances in therapy—a substantial cause of death among critically ill patients [2]. An early diagnosis and evaluation of its severity can be difficult due to varying and sometimes unspecific clinical presentations [3]. Still, early diagnosis and risk evaluation increase the prognosis substantially [4, 5]. Besides clinical assessment and common scores such as the sepsis-related organ failure assessment (SOFA) score, biomarkers may facilitate and even improve diagnosis and risk-adapted treatment in critically ill patients [6].

Growth differentiation factor-15 (GDF-15), also known as macrophage inhibitory cytokine-1 (MIC-1), placental bone morphogenetic protein, placental transforming growth factor, and nonsteroidal anti-inflammatory drug-activated gene-1 [7], is a member of the transforming growth factor-*β* (TGF*β*) superfamily. It is present in most adult tissues and strongly expressed in epithelial cells and macrophages [8]. Additionally, it correlates with macrophage activation [9]. In recent years, circulating GDF-15 has been identified as a biomarker with prognostic value in cardiopulmonary diseases like heart failure [10], ST- and non-ST-elevation myocardial infarction [11–13], and pulmonary embolism [14]. Other investigations reported increased GDF-15 levels correlating with prognosis in end-stage renal disease [15, 16], acute respiratory distress syndrome [17], liver injury [18, 19], and chronic inflammatory diseases such as rheumatoid arthritis [20]. Although the biological significance underlying these findings is largely obscure, GDF15 seems to be an integrative signal in pathologic conditions, providing information on the severity of disease [21].

As all types of organ failure (heart, lung, liver, and kidney) related to high GDF-15 are relevant for the prognosis of sepsis [1], we hypothesized that GDF-15 might represent an interesting prognostic biomarker in critically ill patients with sepsis. However, data on GDF-15 in patients with sepsis are scarce. A recent small study found increased GDF-15 levels in 15 patients with sepsis [22]. Another larger study investigated 530 patients from a medical intensive care unit (ICU), but this study included only 14 patients (2% of the total cohort) with sepsis [23]. We therefore investigated GDF-15 serum concentrations in a large cohort of 219 consecutively enrolled critically ill patients, including 146 subjects with sepsis, in order to identify associations between GDF-15 and organ dysfunction and disease severity, as well as ICU and overall survival in critically ill patients.

## 2. Materials and Methods

### 2.1. Study Design

We obtained written informed consent from each patient, his or her spouse, or the appointed legal guardian. Patients who were expected to have a short-term (<72 h) intensive care treatment for example due to post-interventional observation or acute intoxication were excluded [24]. All other patients were eligible for this study. The current cohort of patients was collected from an ongoing, prospective observational trial in our unit, in which patients were included consecutively. The sample size based on a power analysis was calculated with a minimum of 184 (based on an assumed effect size of 0.5). For the current analysis, we therefore randomly enrolled *n* = 219 patients that had been treated between 2006 and 2011 from the existing biobank. In addition, 66 healthy blood donors with normal values for blood counts, C-reactive protein, and liver enzymes were included as controls, leading to a total sample size of *n* = 285. The long-term course of patients was followed by direct contact to the patient, the patients' relatives, or their primary care physician. We used the Third International Consensus Definitions for Sepsis and Septic Shock as a post hoc definition for sepsis patients; the others were categorized as nonsepsis patients [1].

The study protocol was approved by the local ethics committee and conducted in accordance with the ethical standards laid down in the 1964 Declaration of Helsinki (ethics committee of the University Hospital Aachen, RWTH-University, Aachen, Germany; reference number EK 150/06).

### 2.2. GDF-15 Measurements

Blood samples were collected directly at admission to the ICU. After centrifugation at 4°C for 10 minutes, serum and plasma aliquots of 1 mL were frozen immediately at −80°C. GDF-15 serum concentrations were analysed using a commercially available ELISA (human GDF-15 Quantikine® Immunoassay, number DGD150, R&D Systems, Minneapolis, MN) following the manufacturer's protocol. The scientist performing laboratory measurements was fully blinded to any clinical or other laboratory data of the patients or controls.

### 2.3. Statistical Analysis

Data are given as median and range due to the skewed distribution of most of the parameters. Differences between two groups were assessed by Mann–Whitney *U* test or chi-squared test. Box plot graphics are used to illustrate differences between subgroups. They show a summary of the median, quartiles, range, and extreme values. Their whiskers range from the minimum to the maximum values excluding outliers displayed as separate points. An outlier was defined as a value that is smaller than the lower quartile minus 1.5 times interquartile range or larger than the upper quartile plus 1.5 times the interquartile range. A far out value was defined as a value that is smaller than the lower quartile minus three times interquartile range or larger than the upper quartile plus three times the interquartile range [25]. All values have been included for statistical analyses. Correlations between variables were analysed using the Spearman correlation tests. Parameters correlating with GDF-15 levels at admission were included in a multivariate linear regression analysis with GDF-15 as the dependent variable to find independent (meaningful) predictors of elevated GDF-15. The prognostic value of the variables was tested by univariate and multivariate analysis using the Cox regression model. In order to illustrate differences in survival, Kaplan Meier curves were plotted [26]. Differences between the curves were assessed using the log-rank test. Receiver operating characteristic (ROC) curve analyses were used to assess the value of a predictive marker or a composite score. ROC curves were generated by plotting sensitivity against 1 − specificity [27]. Statistical analyses were performed with SPSS version 23 (SPSS, Chicago, IL, USA).

## 3. Results

### 3.1. GDF-15 Serum Concentrations Are Increased in Critically Ill Patients and Associated with Sepsis

**Figure 1:**
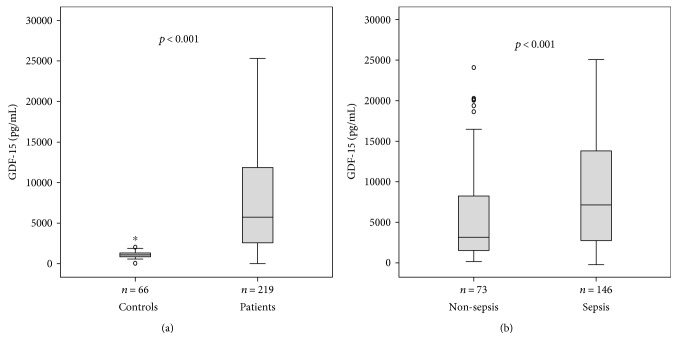
Serum GDF-15 concentrations in critically ill patients and sepsis. (a) Serum levels of GDF-15, at the time of admission to the ICU, were significantly higher in critically ill patients than in healthy controls (*p* < 0.001; *U* test). (b) GDF-15 levels were significantly higher in patients with sepsis (*p* < 0.001) compared to ICU patients without sepsis.

In order to investigate the role of GDF-15 in critical illness, we measured serum levels in 219 patients at the time of admission to our medical ICU prior to therapeutic interventions. In comparison to 66 healthy controls, GDF-15 levels were greatly increased in critically ill patients (median 1097 versus 5753 pg/mL, *p* < 0.001, *U* test; Figure 1(a)).

Among all 219 ICU patients, 146 were admitted because of sepsis including severe sepsis and septic shock. The most common site of infection was pulmonary (*n*, 72), followed by abdominal foci (*n*, 26), and urosepsis (*n*, 11) (detailed data not shown). Nonseptic ICU patients suffered from cardiopulmonary diseases (*n* = 29), pancreatitis (*n* = 13), decompensated liver cirrhosis (*n* = 9), and other nonseptic diseases (*n* = 22). Interestingly, GDF-15 levels were significantly higher in septic compared to nonseptic ICU patients (median 3409 versus 7410 pg/mL; *p* < 0.001; Figure 1(b), Table 1). Sepsis and nonsepsis patients did not differ in age or sex, but sepsis patients had significantly higher APACHE II or SOFA scores and longer periods of mechanical ventilation (Table 1). In accordance with prior reports from our group [26, 28], soluble urokinase plasminogen activator receptor (suPAR) and amino-terminal propeptide of C-type natriuretic peptide (NT-proCNP) were significantly elevated in patients with sepsis (median suPAR 10.7 ng/mL; median NT-proCNP 4.9 pmol/L) as compared with patients without sepsis (median suPAR 5.7 ng/mL, *p* < 0.001; median NT-proCNP 0.9 pmol/L, *p* < 0.001).

To determine the predictive value of GDF-15 in identifying sepsis at admission, we conducted a ROC analysis comparing GDF-15 with classical markers such as procalcitonin and C-reactive protein (CRP). While procalcitonin and CRP reached AUCs of 0.762 (95% CI 0.686–0.838) and 0.836 (95% CI 0.771–0.902), respectively, GDF-15 only achieved an AUC of 0.646 (95% CI 0.556–0.736).

### 3.2. GDF-15 Levels in Critically Ill Patients Correlate with Clinical Scores and Organ Dysfunction

In order to assess a possible association between GDF-15 and organ dysfunction, we performed extensive correlation analyses between levels of GDF-15 and various established and experimental laboratory markers as wells as clinical scores (Table 2). Here, we found strong associations between GDF-15 levels and established markers of renal dysfunction (e.g., creatinine (*r* = 0.481, *p* < 0.001) and cystatin C (*r* = 0.553, *p* < 0.001)), cholestasis (e.g., bilirubin (*r* = 0.195, *p* = 0.004) and *γ*GT (*r* = 0.200, *p* = 0.003)), impaired hepatic synthesis (e.g., albumin (*r* = −0.206, *p* = 0.028), PCHE (*r* = −0.149, *p* = 0.037), and prothrombin time (*r* = −0.314, *p* < 0.001)), and cardiac failure (e.g., brain natriuretic peptide (*r* = 0.278, *p* = 0.024)). Moreover, GDF-15 levels also correlated with markers of general inflammation (e.g., C-reactive protein (CRP) (*r* = 0.268, *p* < 0.001) and interleukin 6 (IL6) (*r* = 0.293, *p* < 0.001)).

**Figure 2:**
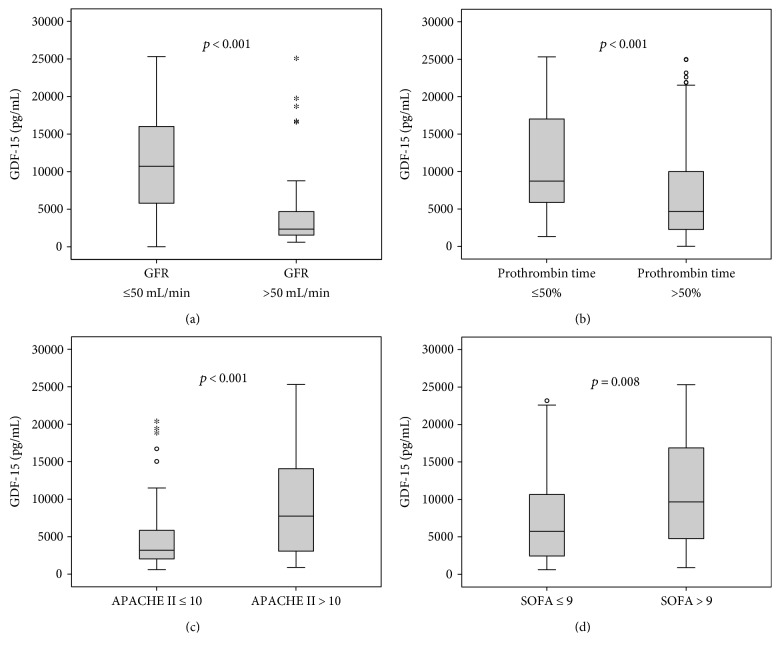
Serum GDF-15 levels in critically ill patients are associated with organ failure and disease severity. Serum levels of GDF-15 at the time of admission to the ICU were significantly higher in critically ill patients with renal failure (cystatin C-based glomerular filtration rate (GFR) < 50 mL/min (a)); asterisks denote significance values at) and hepatic failure (prothrombin time < 50% (b)). Critically ill patients with higher disease severity as represented by clinical scores (APACHE II (c) and SOFA (d)) showed significantly higher GDF-15 levels.

Strikingly, patients with manifest organ failure had significantly elevated GDF-15 serum concentrations. This was observed for patients with renal failure (defined as a cystatin C-based glomerular filtration rate below 50 mL/min, Figure 2(a)) or hepatic dysfunction (defined as prothrombin time < 50%, Figure 2(b)). Similar results were obtained, when hepatic dysfunction was defined as bilirubin levels above 3 g/dL (*p* = 0.031, detailed data not shown). Moreover, GDF-15 levels were associated with disease severity. Patients with higher APACHE II and SOFA scores showed significantly increased levels of GDF-15 in their serum (Figures 2(c) and 2(d)). GDF-15 also positively correlated with these disease severity scores (APACHE II: *r* = 0.349, *p* < 0.001; SOFA: *r* = 0.326, *p* < 0.001; Table 2). These associations were also found in the subgroup of patients with sepsis (Table 2).

### 3.3. GDF-15 at Admission Is an Independent Predictor of ICU Mortality

**Figure 3:**
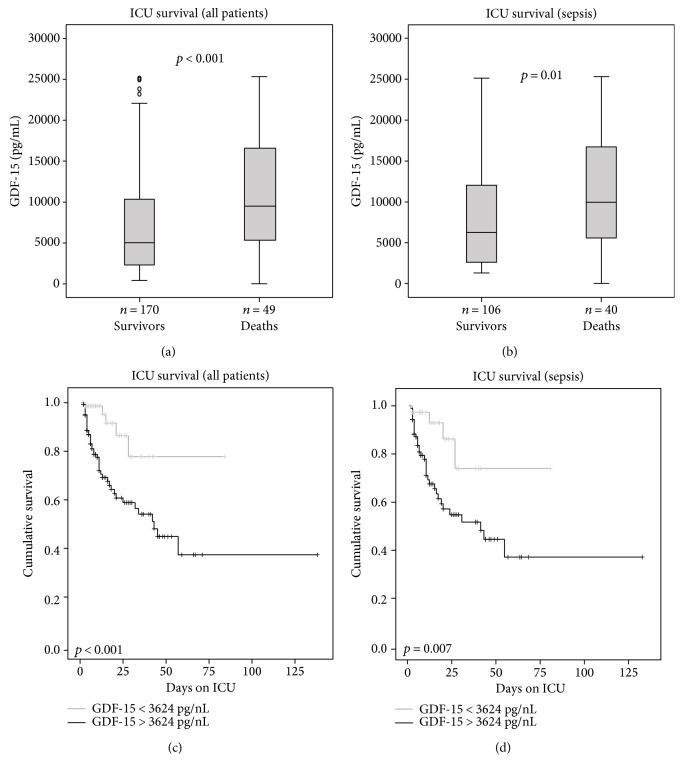
Prediction of ICU mortality by GDF-15 serum levels. (a, b) Patients that died during the course of ICU treatment ((a) all patients; (b) sepsis only) had significantly higher serum GDF-15 levels on admittance to the ICU (*p* < 0.001) than survivors. (c, d) Kaplan-Meier survival curves of ICU patients are displayed, showing that patients with GDF-15 below a cut-off value of 3624 pg/mL had a better outcome at the ICU ((c) all patients; (d) sepsis only).

The strong correlation between different types of organ dysfunction and GDF-15 led us to hypothesize that GDF-15 might predict mortality in critically ill patients as early as at the time of ICU admission. In total, 22.4% of the patients died at the ICU and 40.6% during the whole observation time (of up to three years). Patients with sepsis had an increased ICU (*p* = 0.012) as well as overall mortality (*p* = 0.025) compared to patients without sepsis. Strikingly, patients that survived ICU treatment showed significant lower serum levels of GDF-15 than nonsurvivors (median 5028 versus 9505 pg/mL; *p* < 0.001; Figure 3(a)) in all patients as well as in the subgroup of sepsis patients (median 6244 pg/mL versus 9964 pg/mL; *p* = 0.01; Figure 3(b)).

In order to analyse the practical value of GDF-15 in predicting ICU survival, we used receiver operating characteristic (ROC) curves. Here, we could show that the prognostic accuracy of GDF-15 (AUC 0.672; 95% CI 0.570–0.774) is comparable to the widely used APACHE II score (AUC 0.685; 95% CI 0.576–0.795) and appeared superior to the established marker procalcitonin (AUC 0.592; 95% CI 0.488–0.695).

By Cox regression analysis, high GDF-15 levels were found to predict ICU mortality (*p* = 0.009). Using the Youden index [29] based on the coordinates of the ROC analysis, a GDF-15 cut-off value of 3624 pg/mL revealed the best sensitivity and specificity in predicting ICU mortality. The Kaplan-Meier survival curve analysis confirmed that high GDF-15 levels (>3624 pg/mL) were strongly associated with ICU mortality in all patients (Figure 3(c)) and sepsis patients (Figure 3(d)).

However, as GDF-15 is closely correlated with markers of organ failure and inflammation, we next tested whether GDF-15 serum concentrations can serve as an independent predictor of survival by Cox regression analyses. When we included age, markers of inflammation (i.e., CRP), and renal (i.e., creatinine), circulatory (i.e., lactate), and hepatic dysfunction (i.e., bilirubin and prothrombin time), high GDF-15 was an independent predictor of ICU mortality in critically ill patients with an HR of 3.42 (95% CI 1.33–8.78, Table 3). In the subgroup of sepsis patients, GDF-15 remained independent when assessed together with the same set of covariates with an HR of 3.16 (95% CI 1.10–9.07; Table 3).

### 3.4. High Levels of GDF-15 at Admission Predict Poor Overall Survival

**Figure 4:**
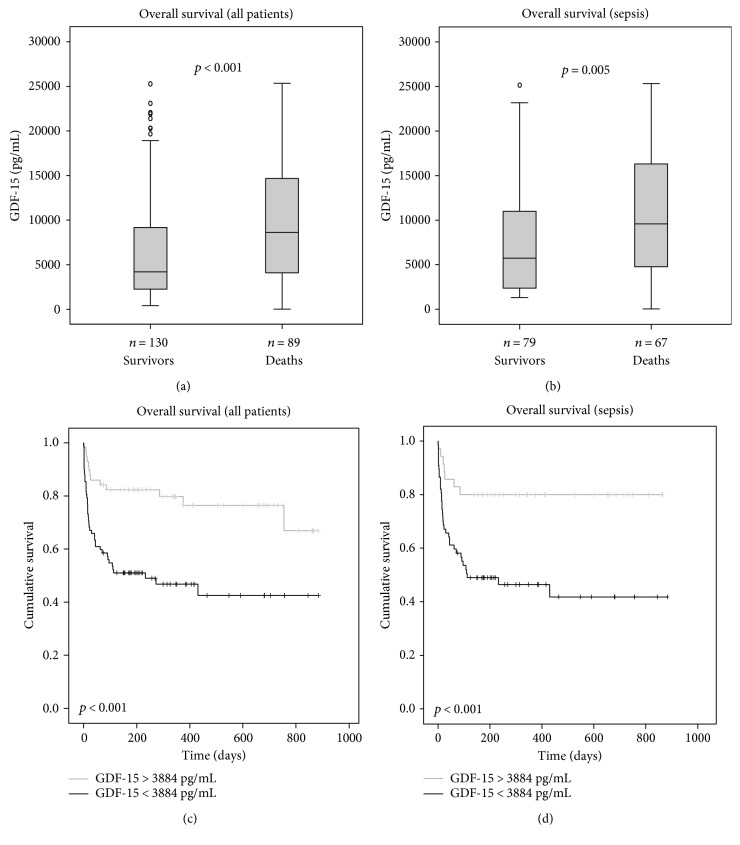
Prediction of overall mortality by GDF-15 serum concentrations. (a, b) Patients that died during the total observation period ((a) all patients; (b) sepsis only) had significantly higher serum GDF-15 levels on admittance to ICU than survivors (*p* < 0.001). (c, d) Kaplan-Meier survival curves of ICU patients are displayed, showing that patients with GDF-15 levels above a cut-off value of 3884 pg/mL have an increased overall mortality ((c) all patients; (d) sepsis only).

Given the excellent prognostic value of GDF-15 in predicting short-term survival, we hypothesized that it might even predict long-term survival. GDF-15 levels were significantly increased in patients that died during the observation period (median 8609 versus 4192 pg/mL; *p* < 0.001; Figure 4(a)). Similar results were seen in the large subgroup of sepsis patients (median 5726 versus 9566 pg/mL; *p* = 0.005; Figure 4(b)).

Again, we used ROC curves to assess the diagnostic quality of GDF-15 in predicting overall mortality in our cohort. Similar to ICU mortality, we found the prognostic accuracy of GDF-15 (AUC 0.630; 95% CI 0.538–0.721) comparable to the APACHE II score (AUC 0.629; 95% CI 0.536–0.722) and higher than procalcitonin (AUC 0.589, 95% CI 0.494–0.683).

In addition, we calculated the Youden index based on the coordinates of the ROC analysis and found an optimal cut-off value of 3884 pg/mL. The Kaplan-Meier survival curve analysis showed an excellent discrimination with regard to overall survival in all critically ill patients (Figure 4(c)) as well as in sepsis patients (Figure 4(d)).

Moreover, we conducted uni- and multivariate Cox regression analyses including age, markers of inflammation (i.e., CRP), and renal (i.e., creatinine), circulatory (i.e., lactate), and hepatic dysfunction (i.e., bilirubin and prothrombin time). GDF-15 remained an independent predictor of overall mortality with an HR of 2.0 (95% CI 1.02–3.88, Table 4). For the large subgroup of sepsis patients, GDF-15 was an independent risk factor for an unfavorable long-term survival, too (HR 2.0; 95% CI 1.02–3.88; Table 4).

## 4. Discussion

Although its biological functions are not fully understood, circulating levels of the inflammation-associated protein GDF-15 increase in conditions of failure of such different organs as the heart [30], liver [19], and kidney [15]. We therefore hypothesized that GDF-15 could be a useful novel biomarker in critically ill patients that might integrate information on multiorgan dysfunction that is prognostically relevant especially in sepsis and septic shock [1]. In fact, two smaller studies on 15 and 14 sepsis patients had already indicated increased levels of GDF-15 at the ICU [22, 23]. Our study comprising 219 patients including 146 sepsis patients revealed (i) highly elevated GDF-15 in ICU patients versus healthy controls (median values about 5-fold increased), (ii) higher levels in sepsis versus nonsepsis patients, (iii) close associations between serum GDF-15 and organ dysfunction as well as inflammation, and (iv) an exceptional value of GDF-15 as a biomarker predicting ICU and overall mortality in critically ill patients, especially with sepsis.

Using a very heterogeneous cohort of 530 consecutive ICU patients including neurological disorders and post-surgery patients, Dieplinger and coworkers suggested high GDF-15 levels as an indicator of increased mortality risk [23]. Interestingly, this study found slightly lower cut-off values for GDF-15 that discriminate patients with favorable or poor prognosis (3470 pg/mL), but GDF-15 was not an independent predictor of mortality in this study [23]. In contrast, our study identified GDF-15 as an independent prognostic biomarker. Possibly, due to the different design of our study (i.e., exclusion of post-surgery/post-intervention ICU patients without critical illness; only medical ICU patients; and high number of patients with sepsis), our cohort represents a more homogeneous population of critically ill patients.

Nevertheless, this study has several limitations. One is the single-center setting from a medical ICU, which does not allow to easily extrapolate to different settings (e.g., surgical and neurological) of critical illness. Moreover, we did not perform longitudinal measurements of GDF15 over the course of the ICU stay. Also, organ failure assessment was conducted with laboratory but no functional or invasive tests (like echocardiography or liver biopsy).

The association between GDF-15 and patients' outcome is by far not limited to the ICU setting but has been described in special patient cohorts and even in the general community. A population-based cohort study from Sweden on 876 male volunteers, aged between 35 and 80 years, found that GDF-15 is associated with all-cause (cardiovascular, cancer, etc.) mortality independent from age. This was confirmed in a parallel twin study and remained independent of the genetic background [31]. In a recent meta-analysis on the prognostic power of GDF-15 in patients with heart failure, circulating GDF-15 levels were a strong prognosticator of all-cause mortality in heart failure patients [32].

The pathogenic mechanisms underlying the prognostic power of GDF-15 remain unclear. Firstly, GDF-15 is induced by proinflammatory cytokines such as tumor necrosis factor and IL6 [9, 21]. Thus, it might reflect proinflammatory processes and macrophage activation [9]. In our cohort, we indeed observed clear associations with sepsis, IL6, CRP, and other inflammation-related biomarkers. Secondly, GDF-15 is increased in experimental conditions of ischemia/reperfusion injury [7] as well as in clinical settings of organ dysfunction like heart failure [10], myocardial infarction [11–13], and pulmonary embolism [14]. Experimental data from GDF-15-deficient mice indicate that the induction and release of GDF-15 might be tissue protective, as it blocks cell death pathways and induces regeneration [7]. These findings might explain the close association of GDF-15 and multiple parameters of organ dysfunction that we observed in our cohort. Moreover, lactate levels, an established marker of impaired macro- and microcirculation [33], closely correlated with GDF-15 in our study. Thirdly, GDF-15 can have pro- or antiproliferative properties in different settings of cancer [34, 35]. Although this is not directly related to the ICU cohort, GDF-15 might be involved in regenerative processes and thereby influence long-term survival.

Due to the diverse and partially opposing functions described for GDF-15 that appear to depend on the state of cells and the microenvironment [21], it is difficult to foresee whether the association of GDF-15 with organ failure and mortality in critically ill patients can be translated into new therapeutic approaches. On the one hand, inhibition of GDF-15 has been suggested to dampen inflammatory reactions and cardiovascular diseases [36]. On the other hand, augmentation of GDF-15 has been suggested as a beneficial approach to acute ischemic tissue injury [37]. Therefore, more experimental and clinical studies are needed to further specify the pathogenic role of GDF-15 in critical illness. Especially, the strong association between GDF-15 and mortality raises the question if it represents a potentially modifiable process and a possible drug target.

## 5. Conclusions

GDF-15 serum concentrations are significantly increased in critically ill patients, especially in sepsis. They are associated with different types of organ failure in critical illness. High levels of GDF-15 predict an unfavorable ICU and overall outcome. Future studies should consider including this biomarker into multiparametric prognostic scores in order to improve their accuracy.

## Figures and Tables

**Table 1:** Baseline patient characteristics and GDF-15 serum measurements.

Parameter	All patients	Sepsis	Nonsepsis	*p* ^∗^
Number	219	146	73	
Sex (male/female)	134/85	86/60	48/25	n.s.
Age median, (range) [years]	64 (18–90)	65 (20–90)	61 (18–85)	n.s.
Charlson comorbidity index, median (range)	2 (0–9)	2 (0–9)	2 (0–6)	n.s.
APACHE II score, median (range)	18 (2–43)	19 (4–43)	13.5 (2–33)	<0.001
SOFA score, median (range)	9 (0–19)	9.5 (2–19)	7 (0–17)	0.002
Mechanical ventilation, *n* (%)	143 (65.3)	97 (66.4)	46 (63)	n.s.
Ventilation time, median (range) [h]	116 (0–3628)	123.5 (0–2966)	66 (0–3628)	n.s.
Vasopressor demand, *n* (%)	200 (91.3)	132 (90.4)	68 (93.2)	n.s.
ICU days, median (range)	7 (1–137)	6 (1–45)	8.5 (1–137)	0.005
Death during ICU, *n* (%)	49 (22.4)	40 (27.4)	9 (12.3)	0.012
Overall mortality, *n* (%)	89 (40.6)	67 (45.9)	22 (30.1)	0.025
GDF-15 day 1, median (range) [pg/mL]	5753 (22.8–25,316)	7410 (22.8–25,316)	3490 (415.8–24,336)	<0.001

For quantitative variables, median and range (in parenthesis) are given. GDF-15: growth differentiation factor-15; APACHE: acute physiology and chronic health evaluation; SOFA: sequential organ failure assessment. ^∗^Significance between sepsis and nonsepsis patients was assessed using the Mann–Whitney *U* test or chi-squared test, respectively.

**Table 2:** Correlations of GDF-15 with clinical scores and other laboratory markers.

	All patients		Sepsis		Nonsepsis	
	*r*	*p*	*r*	*p*	*r*	*p*
*Markers of inflammation*						
CRP	0.268	<0.001	0.137	n.s.	0.256	0.029
Procalcitonin	0.328	0.007	0.277	0.049	0.321	n.s.
IL10	0.266	0.005	0.365	0.004	0.053	n.s.
IL6	0.293	<0.001	0.203	0.032	0.372	0.006
*Markers of organ dysfunction*						
Creatinine	0.481	<0.001	0.301	<0.001	0.373	0.001
Cystatin	0.553	<0.001	0.490	<0.001	0.409	<0.001
GFR	−0.489	<0.001	0.490	<0.001	0.626	<0.001
AST	0.257	<0.001	0.455	<0.001	0.352	0.011
ALT	0.096	n.s.	0.256	0.003	0.370	0.002
GLDH	0.232	0.001	0.076	n.s.	0.297	0.011
Bilirubin	0.195	0.004	0.224	0.012	0.354	0.003
*γ*GT	0.200	0.003	0.14	n.s.	0.304	0.01
PCHE	−0.149	0.037	0.12	n.s.	0.347	0.003
Prothrombin time	−0.314	<0.001	−0.121	n.s.	−0.084	n.s.
Albumin	−0.206	0.028	0.235	0.036	−0.125	n.s.
Urea	0.440	<0.001	0.387	<0.001	0.451	<0.001
Lactate	0.222	0.001	0.333	<0.001	0.111	n.s.
LDH	0.244	<0.001	0.176	0.033	.375	0.001
NT-proBNP	0.278	0.024	0.328	0.019	−0.077	n.s.
Fibrinogen	0.093	n.s.	−0.027	n.s.	0.074	n.s.
*Clinical scores*						
APACHE II	0.349	<0.001	0.363	<0.001	0.177	n.s.
SOFA	0.326	<0.001	0.371	0.001	0.176	n.s.
SAPS2	0.394	0.001	0.554	<0.001	0.168	n.s.
*New and experimental biomarkers*						
APRIL	0.314	<0.001	0.201	0.04	0.409	0.001
Ghrelin	−0.181	n.s.	−0.293	n.s.	−0.013	n.s.
Adiponectin	0.246	0.065	0.192	n.s.	0.279	n.s.
Resistin	0.287	0.031	0.256	n.s.	−0.063	n.s.
Leptin	−0.122	n.s.	−0.135	n.s.	−0.053	n.s.
NT-proCNP	0.431	<0.001	0.549	<0.001	−0.001	n.s.
suPAR	0.441	<0.001	0.368	<0.001	0.444	0.005

ALT: alanine aminotransferase; APACHE: acute physiology and chronic health evaluation score; APRIL: a proliferation-inducing ligand; AST: aspartate aminotransferase; BNP: brain natriuretic peptide; CRP: C-reactive protein; GFR: glomerular filtration rate; *γ*GT: gamma-glutamyl transpeptidase; GLDH: glutamate dehydrogenase; IL10: interleukin 10; IL6: interleukin 6; LDH: lactate dehydrogenase; NT-proCNP: amino-terminal propeptide of C-type natriuretic peptide; PCHE: pseudocholinesterase; SAPS2: simplified acute physiology score; SOFA: sepsis-related organ failure assessment score; suPAR: soluble urokinase plasminogen activator receptor.

**Table 3:** Uni- and multivariate Cox regression analyses for GDF-15 levels at ICU admission to predict ICU mortality.

	All patients				Sepsis patients			
	Unadjusted HR (95% CI)	*p*	Adjusted HR (95% CI)	*p*	Unadjusted HR (95% CI)	*p*	Adjusted HR (95% CI)	*p*
GDF-15 > 3624 pg/mL	4.41 (1.74–11.17)	0.009	3.42 (1.33–8.78)	0.027	3.75 (1.33–10.58)	0.012	3.16 (1.10–9.07)	0.033
Creatinine	—	n.s.	—	n.s.	0.99 (0.97–1.02)	0.046	—	n.s.
CRP	—	n.s.	—	n.s.	—	n.s.	—	n.s.
Bilirubin	—	n.s.	—	n.s.	—	n.s.	—	n.s.
Prothrombin time	—	n.s.	—	n.s.	—	n.s.	—	n.s.
Lactate	1.11 (1.03–1.20)	0.008	1.13 (1.05–1.22)	0.002	1.15 (1.07–1.24)	<0.001	1.12 (1.04–1.22)	0.005
Age	1.03 (1.01–1.05)	0.006	1.03 (1.01–1.05)	0.009	—	n.s.	—	0.009

95% CI: 95% confidence interval; CRP: C-reactive protein; GDF-15: growth differentiation factor-15.

**Table 4:** Uni- and multivariate Cox regression analyses for GDF-15 levels at ICU admission to predict overall mortality.

	All patients				Sepsis patients			
	Unadjusted HR (95% CI)	*p*	Adjusted HR (95% CI)	*p*	Unadjusted HR (95% CI)	*p*	Adjusted HR (95% CI)	*p*
GDF-15> 3884 pg/mL	3.03 (1.63–5.66)	0.005	2.0 (1.02-3.88)	0.041	3.93 (1.66–9.33)	0.002	2.61 (1.14–6.02)	0.033
Creatinine	—	n.s.	—	n.s.	—	n.s.	—	n.s.
CRP	—	n.s.	—	n.s.	—	n.s.	—	n.s.
Bilirubin	1.20 (1.03–1.40)	0.023	—	n.s.	—	n.s.	—	n.s.
Prothrombin time	0.99 (0.98–0.99)	0.037	—	n.s.	—	n.s.	—	n.s.
Lactate	—	n.s.	—	n.s.	1.21 (1.01–1.45)	0.04	—	n.s.
Age	1.03 (1.01–1-05)	0.004	1.028 (1.01–1.05)	0.017	1.03 (1.01–1.06)	0.008	—	n.s.

95% CI: 95% confidence interval; CRP: C-reactive protein; GDF-15: growth differentiation factor-15.
